# Associations of Serum S100A12 With Severity and Prognosis in Patients With Community-Acquired Pneumonia: A Prospective Cohort Study

**DOI:** 10.3389/fimmu.2021.714026

**Published:** 2021-10-22

**Authors:** Xiao Jiang, Chun-Mei Huang, Chun-Mei Feng, Zheng Xu, Lin Fu, Xin-Ming Wang

**Affiliations:** ^1^ Department of Nephrology, First Affiliated Hospital of Anhui Medical University, Hefei, China; ^2^ Anhui Province Key Laboratory of Major Autoimmune Diseases, Anhui Institute of Innovative Drugs, School of Pharmacy, Anhui Medical University, Hefei, China; ^3^ Respiratory and Critical Care Medicine, Second Affiliated Hospital of Anhui Medical University, Hefei, China; ^4^ Department of Toxicology, Anhui Medical University, Hefei, China; ^5^ Department of Pharmacy, First Affiliated Hospital of Anhui Medical University, Hefei, China; ^6^ Third-Grade Pharmaceutical Chemistry Laboratory of State Administration of Traditional Chinese Medicine, Hefei, China

**Keywords:** S100A12, community-acquired pneumonia, CAP severity score, prognosis, biomarker

## Abstract

**Background:**

Previous studies indicated the calcium-binding protein S100A12 to be involved in the pathophysiology of pulmonary inflammatory diseases. However, the role of S100A12 has remained elusive in patients with community-acquired pneumonia (CAP). Therefore, the purpose of this prospective cohort study was to evaluate the association between serum S100A12 with severity and prognosis in CAP patients.

**Methods:**

Two groups with either 239 CAP patients or 239 healthy controls were enrolled in our study. Fasting blood and clinical characteristics were collected. On admission, serum S100A12 was measured using enzyme-linked immunosorbent assay (ELISA).

**Results:**

Serum S100A12 was increased in CAP patients compared to control subjects. Furthermore, serum S100A12 was elevated according to the severity of CAP. Correlative analysis suggested that the level of serum S100A12 was associated with blood routine indices, renal function markers, inflammatory cytokines and other clinical parameters among CAP patients. Additionally, linear and logistical regression analyses indicated that serum S100A12 was positively associated with CAP severity scores in CAP patients. In addition, the association of high serum S100A12 and prognosis was accessed using a follow-up research. Elevated serum S100A12 on admission increased the risk of death and hospital stay in CAP patients during hospitalization.

**Conclusions:**

Elevated serum S100A12 on admission is positively associated with the severity and adverse prognosis in CAP patients, suggesting that S100A12 may involve in the pathophysiological process of CAP. The titre of serum S100A12 may be used as a biomarker for diagnosis and prognosis among CAP patients.

## Introduction

Community-acquired pneumonia (CAP) which is predominately caused by *Steptococcus pneumoniae* is one of the most widespread and fatal infectious pathologies. It accounts for approximately 3 million deaths per year worldwide (1). The annual incidence of CAP diagnosed varies between different communities and ranges from five to 11 cases per 1,000 persons. The incidence of CAP needing hospitalization is highest among elderly people and adults hospitalized with CAP display a mortality between 5.7% and 14% (2, 3). In the USA, CAP causes 1.2 million hospitalizations, 2.3 million emergency treatments, and evokes a huge economic burden for individuals and society with total costs of more than $10 billion every year (4, 5). Albeit new drugs have been made available and new therapies are used in clinical practice, the rate of death is still high (6, 7). Therefore, timely and accurate evaluation of the disease severity is crucial to stratify patients and to reduce the mortality among the most vulnerable CAP patients.

The calcium-binding protein A12 that belongs to the S100 family of low molecular weight proteins (S100A12), is also called calgranulin C and consists of 92 amino acids that form an EF-hand calcium-binding motif that consists of helix-loop-helix structures connected by a central hinge region (8, 9). S100A12 is present in the cytoplasm of myeloid cells such as neutrophils, lymphocytes, and monocytes (10, 11) and is involved in multiple cellular activities including chemotactic activity, inflammatory reaction, oxidative stress, and activation of intracellular signaling cascades, all of which have been implicated in the innate immune response and certain autoimmune reactions (12, 13). As we all know, the host immune response to infection changes across the life course, both in the very young and with age and frailty (14). Several studies indicated that immune status is always not significantly changed in mild CAP patients. However, immune status is obviously repressed in severe CAP patients. Neutrophils are increased, and lymphocytes are reduced in severe CAP patients. Moreover, human T cells in the early control and NK cells in the late control are recruited to the lungs to eliminate pathogens after infection in the process of CAP. Increasing evidences have demonstrated that the counts of immune cells, such as B cells, T cells, and NK cells, were strongly correlated with the severity in CAP patients (15–17). Previous studies suggested that an increase of S100A12 is associated with several inflammatory diseases, such as inflammatory bowel disease, rheumatoid arthritis, juvenile idiopathic arthritis, cystic fibrosis, and periodontitis (18–21). Moreover, recent researches indicated that S100A12 can be implicated in idiopathic pulmonary fibrosis, acute lung injury, pulmonary hypertension, interstitial lung disease, and acute respiratory distress syndrome (22–27). These findings allow to speculate that S100A12 could contribute the pathological process of pulmonary inflammatory diseases.

It is widely recognized that CAP is an inflammatory disease. Nevertheless, the role of S100A12 in CAP was unclear at present. It can be reasonably presumed that S100A12 plays a role in the process of CAP and its specific role has been unclear. Therefore, the purpose of this research was to assess a potential correlation of the level of serum S100A12 with severity and prognosis in CAP patients through a prospective cohort study.

## Methods

### Study Design and Subjects

All patients with CAP were enrolled in the Second Affiliated Hospital of Anhui Medical University from June 2019 to March 2021. At the beginning of the project, 309 patients with CAP were eligible and willing to take part in the follow-up research. Finally, 239 CAP patients were recruited after 70 cases were excluded because 35 cases were without complete data, 15 cases withdrew, and serum samples of 20 cases were unavailable. All enrolled cases met the diagnostic criteria of CAP (28) and had not been admitted to the hospital over the past 6 months. The exclusion criteria for all participators were as follows: (1) pregnancy, (2) under 18 years of age, (3) mental health issues, (4) malignances or pulmonary tuberculosis, and (5) immunosuppression. Age- and sex-matched healthy controls were recruited from the physical examination center of the Second Affiliated Hospital. On admission or recruitment (controls), serum was collected from each participant for a laboratory examination before therapeutic intervention. The severity was evaluated with CAP severity scores in CAP patients, such as Pneumonia Severity Index (PSI), CURXO, CURB-65, CRB-65, and SMART-COP. Demographic information and laboratory results were collected from electronic medical record system. This study was approved by the Ethics Committee of Second Affiliated Hospital of Anhui Medical University (YX2021-085) and met the principles stated in the Declaration of Helsinki. Informed consent was obtained from all patients and control subjects.

### Enzyme-Linked Immunosorbent Assay

Serum samples were centrifuged and stored at −80°C. Enzyme-linked immunosorbent assay (ELISA) kits specific for IL-6 (JYM1942Hu) and TNF-α (JYM0110Hu) were obtained from Wuhan ColorfulGene Biological Technology Co. MIP-2 (CSB-E07420h) and S100A12 (CSB-E13095h) ELISA kits were purchased from Cusabio, Wuhan, China (https://www.cusabio.com/). Inflammatory cytokines were detected in the collected sera based on the manufacturer’s instructions with minor adjustments (29, 30).

### Statistical Analysis

All statistical analysis was performed with the Statistical Package of Social Sciences (SPSS) software (version 18.0). Normally distributed continuous variables were expressed as a mean, and nonnormally distributed data were expressed using the median. Categorical variables were represented *via* frequency. To compare differences of demographic information and laboratory results between CAP patients and control subjects, independent sample *t*-test, nonparametric test, or Mann-Whitney *U* test was applied. The correlation between serum S100A12 and clinical parameters were analyzed using Spearman analysis and Pearson analysis. The associations between serum S100A12 and CAP severity scores were evaluated through linear regression analysis and logistical regression analysis. The risk of serum S100A12 elevation on prognosis was explored through logistical regression analysis. A *p*-value of ≤0.05 was considered to be statistically significant.

## Results

### Demographics and Clinical Information

Demographic and clinical details of recruited patients and control subjects were analyzed. As shown in **Table 1**, there was no significant statistical difference of age, BMI, and systolic and diastolic blood pressure between CAP patients and control subjects. The number of study participants with hypertension, diabetes mellitus, and other chronic diseases was increased in CAP patients as compared with the control subjects. The median hospital stay of CAP patients was 10.0 days. During hospitalization, 70 (29.3%) patients were admitted to the ICU, 66 (27.6%) patients underwent mechanical ventilation, 34 (14.2%) patients were treated with vasoactive agents, and 22 (9.2%) patients died. Overall, the severity of CAP was evaluated using CAP severity scores, such as CURB-65, CRB-65, PSI, CURXO, SMART-COP, and Acute Physiology and Chronic Health Evaluation II (APACHE II).

**Table 1:** Demographic characteristics of participators at baseline.

Variables	CAP (n = 239)	Control (n = 239)	p
Age (years)	62.3 ± 1.32	61.6 ± 2.35	0.351
Male (n (%))	143 (59.8)	155 (64.9)	0.256
BMI	22.7 ± 0.36	23.6 ± 0.46	0.089
Systolic pressure (mmHg)	123.9 ± 2.34	119.8 ± 3.65	0.125
Diastolic pressure (mmHg)	76.8 ± 1.32	75.6 ± 2.31	0.097
Comorbidities			
Hypertension (n (%))	64 (26.8)	21 (8.79)	<0.001
Diabetes mellitus (n (%))	22 (9.2)	6 (2.51)	0.002
Cerebral infarction (n (%))	20 (8.4)	0	<0.001
Coronary heart disease (n (%))	11 (4.6)	0	0.001
Bronchitis (n (%))	19 (7.9)	0	<0.001
Other disease (n (%))	78 (32.6)	11 (4.60)	<0.001
Hospital stay (day)	10.0 (7.0, 17.0)	N.A.	N.A.
ICU admission (n (%))	70 (29.3)	N.A.	N.A.
Mechanical ventilation (n (%))	66 (27.6)	N.A.	N.A.
Vasoactive agent (n (%))	34 (14.2)	N.A.	N.A.
Death (n (%))	22 (9.2)	N.A.	N.A.
CURB-65	1.0 (0, 2.0)	N.A.	N.A.
CRB-65	1.0 (0, 2.0)	N.A.	N.A.
PSI	72.0 (53.0, 97.0)	N.A.	N.A.
CURXO (severe, n (%))	66 (27.6)	N.A.	N.A.
SMART-COP	1.0 (0, 3.0)	N.A.	N.A.
APACHE II	6.0 (4.0, 10.0)	N.A.	N.A.

N.A., not available.

### The Level of Serum S100A12 in CAP Patients and Control Cases

**Figure 1:**
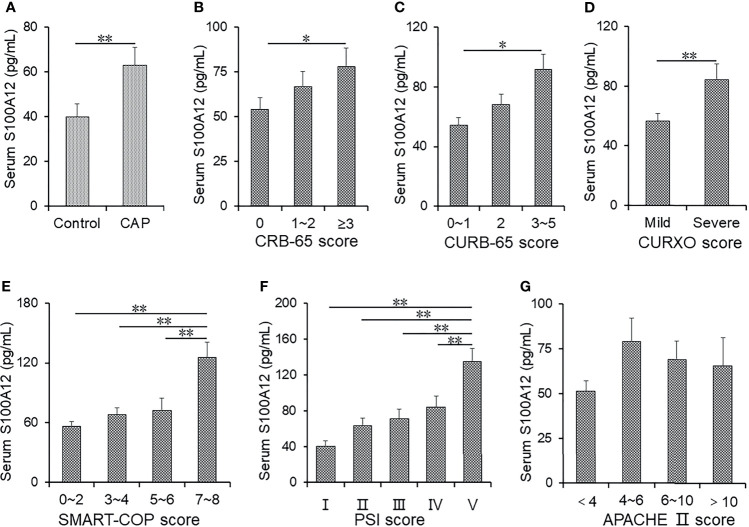
The levels of serum S100A12 in control groups and CAP patients. **(A–G)** The level of serum S100A12 was measured with ELISA. **(A)** The level of serum S100A12 in CAP patients and control cases. **(B)** The level of serum S100A12 in different CRB-65 score of CAP patients. **(C)** The level of serum S100A12 in different CURB-65 score of CAP patients. **(D)** The level of serum S100A12 in different CURXO scores of CAP patients. **(E)** The level of serum S100A12 in different SMART-COP scores of CAP patients. **(F)** The level of serum S100A12 in different PSI scores of CAP patients. **(G)** The level of serum S100A12 in different APACHE II scores of CAP patients. Data were expressed as mean ± SEM. ^*^ *p* < 0.05, ^**^ *p* < 0.01.

The titre of S100A12 was measured in serum using ELISA. As shown in **Figure 1A**, the presence of S100A12 was higher in CAP patients than in healthy cases. According to the CRB-65 score, S100A12 was increased in a patient score higher than 3 compared with a score of 0 in healthy controls (**Figure 1B**) and higher in patients with a score of 3~5 than 0~1 (**Figure 1C**). Additionally, the level of S100A12 was elevated in patients with severe CAP as compared with mild cases (**Figure 1D**). According to SMART-COP score, the level of serum S100A12 was highest at a score of 7~8 (**Figure 1E**). Moreover, the level of serum S100A12 was higher in the grade of V than in other scores (**Figure 1F**). Using APACHE II, there was no difference of serum S100A12 among the patients with different scores as shown in **Figure 1G**.

### Correlations of S100A12 With Clinical Parameters Among CAP Patients

The correlations between serum S100A12 and blood routine indices were analyzed among CAP patients. As shown in **Table 2**, S100A12 was positively associated with the counts of white blood cell (WBC) (*r* = 0.221, *p* < 0.001), neutrophil (*r* = 0.246, *p* < 0.001), and monocyte numbers (*r* = 0.133, *p* = 0.024). Additionally, serum S100A12 was negatively associated with the number of lymphocyte (*r* = −0.223, *p* = 0.046). Moreover, the associations were tested between S100A12 with the indicators of liver, renal, and myocardial functions, as shown in **Table 2**. While there was no association between serum S100A12 with liver function and myocardial function, the level of S100A12 was positively associated with the renal parameters of urea nitrogen (*r* = 0.122, *p* = 0.035) and creatinine (*r* = 0.342, *p* < 0.001) in CAP patients. Moreover, serum S100A12 titre was positively associated with D-dimer (*r* = 0.128, *p* = 0.029), brain natriuretic peptide (BNP) (*r* = 0.200, *p* = 0.016), platelet (PLT) (*r* = 0.142, *p* = 0.017), procalcitonin (PCT) (*r* = 0.310, *p* < 0.001), and fibrinogen (FIB) (*r* = 0.243, *p* < 0.001). Furthermore, S100A12 level was positively associated with inflammatory cytokines, such as tumor necrosis factor-alpha (TNF-α) (*r* = 0.356, *p* = 0.002), macrophage inflammatory protein-2 (MIP-2) (*r* = 0.328, *p* = 0.017), interleukin-6 (IL-6) (*r* = 0.322, *p* = 0.021), and C-reactive protein (CRP) (*r* = 0.262, *p* < 0.001) (**Table 2**).

**Table 2:** Associations between serum S100A12 and clinical characteristics in CAP patients.

Variables	WBC	Neutrophil	Lymphocyte	Monocyte	Eosinophil	Basophil
*r*	0.221	0.246	−0.223	0.133	−0.077	−0.013
*p*	<0.001	<0.001	0.046	0.024	0.126	0.423
**Variables**	**Uric acid**	**Urea nitrogen**	**Creatinine**	**ALT**	**AST**	**CK**
*r*	−0.104	0.122	0.342	0.083	0.028	−0.016
*p*	0.062	0.035	0.001	0.110	0.338	0.422
**Variables**	**CKMB**	**LDH**	**D-Dimer**	**PT**	**BNP**	**PLT**
*r*	−0.006	0.041	0.128	−0.039	0.200	0.142
*p*	0.472	0.312	0.029	0.284	0.016	0.017
**Variables**	**PCT**	**FIB**	**TNF-α**	**MIP-2**	**IL-6**	**CRP**
*r*	0.310	0.243	0.356	0.328	0.322	0.262
*p*	<0.001	<0.001	0.002	0.017	0.021	<0.001

WBC, white blood cell; ALT, alanine aminotransferase; AST, aspartate aminotransferase; CK, creatine kinase; CKMB, creatine kinase isoenzyme; LDH, lactate dehydrogenase; PT, prothrombin; BNP, brain natriuretic peptide; PLT, platelet; PCT, procalcitonin; FIB, fibrinogen; TNF-α, tumor necrosis factor alpha; MIP-2, macrophage inflammatory protein-2; IL-6, interleukin-6; CRP, C-reactive protein.

### Associations Between S100A12 and the Severity in CAP Patients

The correlations between elevated serum S100A12 and different CAP severity scores were analyzed. As shown in **Table 3**, univariable linear regression analysis indicated that the level of serum S100A12 was positively correlated with CRB-65 (*β* = 0.166, 95% CI: 2.960~25.411), CURB-65 (*β* = 0.247, 95% CI: 8.462~26.931), SMART-COP (*β* = 0.263, 95% CI: 5.230~15.150), PSI (*β* = 0.181, 95% CI: 0.119~0.742), and APACHE II (*β* = 0.152, 95% CI: 0.271~3.743). Additionally, univariable logistical regression analysis indicated that serum level of S100A12 was positively correlated with CURXO (OR = 1.006, 95% CI: 1.002~1.009) (**Table 3**). In order to eliminate confounding factors, multivariable linear and logistical regression analyses were performed. After adjusting for age and sex, the titre of serum S100A12 was positively correlated with SMART-COP (*β* = 0.223, 95% CI: 3.331~13.925) and CURXO (OR = 1.004, 95% CI: 1.001~1.008) (**Table 3**).

**Table 3:** Associations between serum S100A12 and CAP severity scores in CAP patients.

	Univariable	*p*	Multivariable^a^	*p*
	*β* (95% CI)		*β* (95% CI)	
CRB-65	0.166 (2.960, 25.411)	0.013	0.099 (−3.873, 20.856)	0.177
CURB-65	0.247 (8.462, 26.931)	<0.001	0.199 (3.986, 24.520)	0.077
SMART-COP	0.263 (5.230, 15.150)	<0.001	0.223 (3.331, 13.925)	0.002
PSI	0.181 (0.119, 0.742)	0.007	0.096 (−0.166, 0.621)	0.255
APACHE II	0.152 (0.271, 3.743)	0.024	0.083 (−0.835, 3.030)	0.264
	OR (95% CI)		OR (95% CI)	
CURXO	1.006 (1.002, 1.009)	0.004	1.004 (1.001, 1.008)	0.022

a Adjusted for age and sex.

### Associations Between S100A12 and the Prognosis in CAP Patients

**Figure 2:**
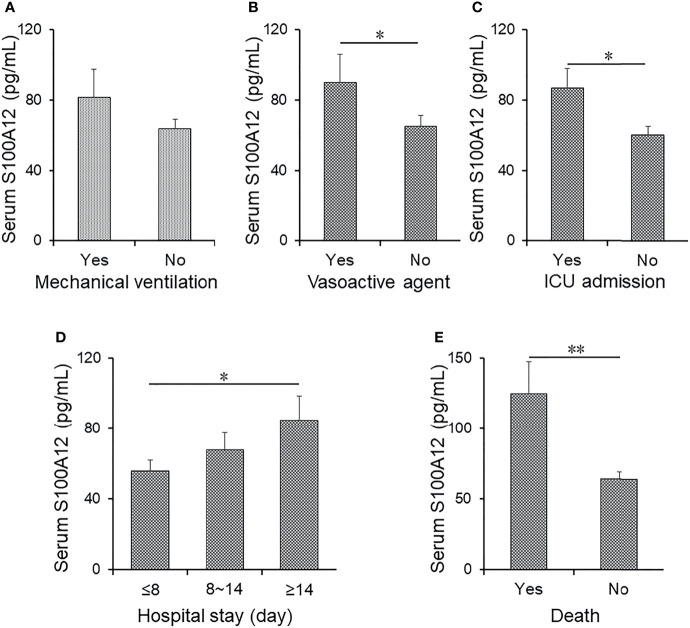
The levels of serum S100A12 in different prognostic outcomes of CAP patients. **(A–E)** The level of serum S100A12 was detected *via* ELISA in CAP patients. **(A)** The level of serum S100A12 in CAP patients with or without mechanical ventilation. **(B)** The level of serum S100A12 in CAP patients with or without vasoactive agent. **(C)** The level of serum S100A12 in CAP patients with or without ICU admission. **(D)** The level of serum S100A12 in CAP patients with different hospital stays. **(E)** The level of serum S100A12 in alive and dead CAP patients. Data were expressed as mean ± SEM. ^*^ *p* < 0.05, ^**^ *p* < 0.01.

Serum level of S100A12 was compared among CAP patients with different prognostic outcomes. As shown in **Figures 2A–C**, serum S100A12 was elevated in CAP patients with mechanical ventilation, vasoactive agents, and ICU admission as compared with those without the abovementioned adverse prognostic outcome. Furthermore, the levels of S100A12 were further compared in CAP patients with different lengths of stay in a hospital. As shown in **Figure 2D**, S100A12 was elevated in patients with stays longer than 14 days as compared with those with stays shorter than 8 days. Finally, the level of serum S100A12 was increased after patients had died from CAP (**Figure 2E**). Taken together, while there was no correlation between serum S100A12 with mechanical ventilation and vasoactive agent, univariable logistical regression analysis found that serum S100A12 was positively correlated with ICU admission (OR = 1.014, 95% CI: 1.011~1.019), death (OR = 1.015, 95% CI: 1.011~1.019), and ≥14 days hospital stay (OR = 1.015, 95% CI: 1.010~1.019) (**Table 4**). Further adjustment for age and sex revealed in multivariable logistical regression analysis that serum S100A12 was positively correlated with death (OR = 1.014, 95% CI: 1.011~1.020) and ≥14 days hospital stay (OR = 1.014, 95% CI: 1.011~1.021).

**Table 4:** Association between serum S100A12 and prognosis in CAP patients.

	Univariable (95% CI)	*p*	Multivariable (95% CI)^a^	*p*
ICU admission	1.014 (1.011, 1.019)	0.049	1.002 (0.999, 1.006)	0.193
Mechanical ventilation	0.997 (0.994, 1.001)	0.147	0.999 (0.995, 1.002)	0.434
Vasoactive agent	1.003 (0.999, 1.007)	0.125	1.002 (0.998, 1.006)	0.314
Death	1.015 (1.011, 1.019)	0.017	1.014 (1.010, 1.019)	0.046
Hospital stay				
≤8	–	1	–	1
8~14	1.003 (0.998, 1.007)	0.278	1.002 (0.997, 1.007)	0.354
≥14	1.015 (1.011, 1.020)	0.033	1.014 (1.011, 1.021)	0.041

a Adjusted for age and sex.

### The Predictive Capacities for Severity and Death Among CAP Patients

**Figure 3:**
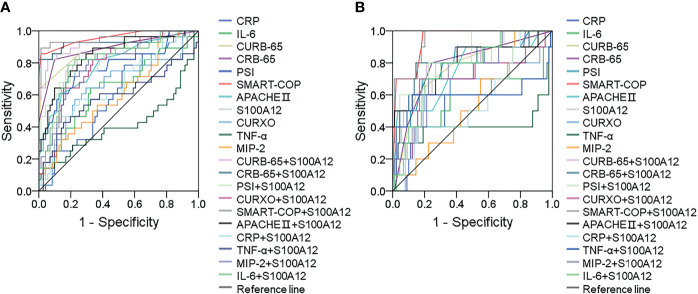
Receiver operating characteristic curves for different predictive biomarkers on admission. **(A)** ROC curve was used to evaluate the predictive values for severity of different biomarkers among CAP patients. **(B)** ROC curve was used to evaluate the predictive values for death of different biomarkers among CAP patients.

The predictive capacities for severity and death between serum S100A12 and CAP severity scores were evaluated using receiver operating characteristic (ROC) area under the curve (AUC). As shown in **Figure 3A** and **Supplementary Table S1**, the predictive capacities for severity of single indices were as follows: SMART-COP score, 0.947; CRB-65 score, 0.889; CURB-65 score, 0.884; serum S100A12, 0.802; CURXO score: 0.795; APACHE II score, 0.795; PSI score, 0.778; serum IL-6, 0.638; serum MIP-2: 0.602; and serum TNF-α: 0.412. Moreover, the predictive capacities for severity of serum S100A12 in combination with CAP severity and inflammatory cytokines were evaluated in CAP patients. The results indicated serum S100A12 in combination with CAP severity scores elevated the predictive capacities of single serum S100A12 and CAP severity scores in CAP patients (**Figure 3A** and **Supplementary Table S1**). Although there was no difference of predictive capacities for death between serum S100A12 and CAP severity scores, the predictive capacities for severity were higher in serum S100A12 and CAP severity scores compared with serum inflammatory cytokines. Besides, the predictive capacities for death were accessed among different biomarkers in CAP patients. The results indicated that the predictive capacities for death were higher in serum S100A12 and CAP severity scores than in inflammatory cytokines. Meanwhile, using serum S100A12 and CAP severity scores together increased the predictive capacities for death than those in single serum S100A12 and CAP severity scores among CAP patients (**Figure 3B** and **Supplementary Table S1**).

## Discussion

This study mainly analyzed the associations of the serum level of S100A12 with severity and prognosis of CAP patients through a prospective cohort study. This study found that: (1) serum S100A12 was elevated in CAP patients; (2) serum S100A12 was gradually elevated in line with the severity of CAP; (3) serum S100A12 was positively correlated with CAP severity scores in CAP patients; and (4) serum S100A12 detectable on admission was positively correlated with death and length of hospital stay in CAP patients.

Previous studies found that S100A12 has different functions including chemotactic activity, inflammatory reaction, oxidative stress, and activation of intracellular signaling cascades. Mounting evidence clarified that S100A12 is elevated and involved in several inflammatory diseases, such as inflammatory bowel disease, rheumatoid arthritis, juvenile idiopathic arthritis, cystic fibrosis, and periodontitis (18–21). According to recently published studies, S100A12 has been implicated in the process of many pulmonary inflammatory diseases (22–27). However, the level of S100A12 was unclear in CAP patients. Consequently, we measured the level of S100A12 in serum between control subjects and CAP patients stratified for disease severity. Our results showed that serum S100A12 was elevated in CAP patients on admission and gradually increased in line with the severity of CAP as shown by different severity scores. Logistical regression analysis further supported this finding. These results suggest that serum S100A12 is positively associated with the severity among CAP patients.

Previous observational studies found that inflammation exerts a key role in the process of CAP (7, 31). Therefore, we evaluated the association between serum S100A12 and inflammatory cytokines and found a positive correlation. Several previous studies from our team had revealed that lymphocyte reduction, liver dysfunction, renal dysfunction, and myocardial injury were observed in patients with Coronavirus disease 2019 (32–35). Publishing data have revealed that *Streptococcus pneumoniae* or other pathogen infection rapidly initiated an inflammatory response. The secretion of inflammatory cytokines and chemokines contributed to attraction of additional innate immune cells such as neutrophils, NK cells, lymphocytes, and monocytes to the site of infection (36, 37). Moreover, hypoinflammatory response always led to lymphocyte reduction and T-cell exhaustion (38). In addition, inflammatory cytokines and chemokines promoting immune cell apoptosis have now been confirmed in many infectious diseases through elevating reactive oxygen species or endoplasmic reticulum stress (39, 40). Animal experiment has revealed that influenza a virus and *Streptococcus* pneumonia coinfection enhanced B-lymphocyte depression and reduction *via* causing lymphocyte apoptosis (41). Therefore, clinical characteristics were measured and analyzed in CAP patients. Lymphocytopenia was observed in CAP patients. Serum S100A12 was inversely associated with lymphocyte and positively associated with renal function in CAP patients. These results are consistent with the findings of previous reports. Earlier research had reported that serum S100A12 was increased and associated with 30-day mortality in patients with acute intracerebral hemorrhage (42). This research implied that serum S100A12 may be useful as a prognostic biomarker of inflammatory diseases. Hence, the association of serum S100A12 on admission and the prognosis in CAP patients was evaluated. The results indicated that serum S100A12 on admission was increased in CAP patients and correlated with vasoactive agent therapy, ICU admission, longer hospital stay, and death during hospitalization. Further logistical regression analysis indicated that serum higher S100A12 on admission elevated the risk of death and longer hospital stay. Moreover, using serum S100A12 and CAP severity scores together increased the predictive capacities for severity and death than those in single serum S100A12 and CAP severity scores in CAP patients. These results suggest that serum S100A12 elevation is positively associated with the adverse prognosis and may be regarded as a prognostic biomarker of CAP.

S100A12 is a proinflammatory alarmin and presents in the cytoplasm of myeloid cells, neutrophils, lymphocytes, and monocytes, as well as structural cells, including endothelial, epithelial, and smooth muscle cells (10, 11). Under physiological conditions, there is sufficient storage of S100A12 in neutrophils and myeloid cells, while S100A12 is significantly elevated during trauma, infection, heat, stress, and many other inflammatory diseases. Infection-induced inflammation is one of the primary resources for S100A12 production. After being infected, the immune system is activated and the number of neutrophils is increasing in human bodies. Next, neutrophils, macrophages, and monocytes produce and secrete S100A12 to circulatory system. Mounting evidences have demonstrated that increasing S100A12 always regulates inflammatory processes, along with evoking the production of inflammatory cytokines, reactive oxygen species, and nitric oxide (43, 44). Moreover, the life span of neutrophils is very short and the death and necrosis of neutrophils also induce the elevation of S100A12 in human bodies (35). An *in vivo* experiment indicated that the level of S100A12 mRNA is increased in human middle ear epithelium-exposed *Streptococcus* pneumonia (45). After S100A12 release, it can bind to toll-like receptor 4 (TLR4) and advanced glycation end product (RAGE) receptor, which is chemotactic for leukocytes and evokes a robust inflammatory reaction in monocytes (46, 47). This binding activates nuclear factor kappa-B (NF-κB), nicotinamide adenine dinucleotide phosphate (NADPH), and mitogen-activated protein kinase (MAPK) pathways, which lead to the production of proinflammatory cytokines and reactive oxygen species (48–51). The production of proinflammatory cytokines and reactive oxygen species further activates a positive feedback loop through recruiting more neutrophils and monocytes. It is possible that the infection of *Streptococcus pneumoniae*, pathogenic bacteria, pathogens infection, or other pathogenic bacteria, activated the immune system and evoked the elevation of neutrophils and production of S100A12 in human bodies. Elevation of S100A12 further activates inflammatory signaling pathways and causes secretion of inflammatory cytokines. In turn, inflammatory cytokine secretion persistently induces the elevation of S100A12 in CAP patients. Therefore, we hypothesize that S100A12 is involve in the pathophysiological process of CAP. Of course, we cannot exclude other ways that pathogenic microbe-induced infection results in the secretion of S100A12 in CAP patients.

While these results suggest that S100A12 exerts a proinflammatory cytokine effect in the process of CAP, we acknowledge that there were several limitations in this study. First, this research was a single-center study with a relatively small sample size. To advance our observations, a multicenter research with larger sample size will be needed. Second, because this study was only a prospective cohort study based on CAP patients, we cannot identify the exact mechanism of S100A12 elevation in CAP. More *in vitro* and *in vivo* experiments will help to eliminate this problem. Third, a replication cohort was lacking; it is difficult for us to perform additional longitudinal research in a short time. Fourth, the level of S100A12 was merely detected in the serum; S100A12 should be determined in the bronchoalveolar lavage fluid of CAP patients in the future. Fifth, a further study will need stratification of the cohort along the pathogens causing CAP to identify further potentially confounding factors.

## Conclusions

In brief, this study mainly explored the associations between serum S100A12 with the severity and prognosis among CAP patients using a prospective cohort study. We found that serum S100A12 is elevated among CAP patients on admission. Serum S100A12 is gradually increased in parallel with the severity in CAP patients on admission. Serum higher S100A12 is positively associated with the severity and adverse prognosis in CAP patients, suggesting that S100A12 may involve in the pathophysiology of CAP. Therefore, these results indicate that serum S100A12 may be used as a biomarker in the diagnosis and prognosis for CAP patients.

## Data Availability Statement

The original contributions presented in the study are included in the article/**Supplementary Material**. Further inquiries can be directed to the corresponding authors.

## Ethics Statement

Written informed consent was obtained from the individual(s) for the publication of any potentially identifiable images or data included in this article.

## Author Contributions

Concept and design of study: LF and X-MW. Acquisition of data: XJ, C-MH, C-MF, and ZX. Analysis of data: XJ, C-MH, C-MF, and ZX. Drafting of manuscript: LF and X-MW. All authors contributed to the article and approved the submitted version.

## Funding

This work was supported by the National Natural Science Foundation of China (82104185, 82100078), the Anhui Provincial Natural Science Foundation (2008085QH400), the open fund of the Key Laboratory of Anti-inflammatory and Immune Medicine, Ministry of Education, P.R. China (Anhui Medical university, KFJJ-2020-03), the Clinical Science Foundation of Anhui Medical university (2021xkj148), National Natural Science Foundation Incubation Program of the Second Affiliated Hospital of Anhui Medical University (2020GQFY05) and Scientific Research of Health Commission in Anhui Province (AHWJ2021b091).

## Conflict of Interest

The authors declare that the research was conducted in the absence of any commercial or financial relationships that could be construed as a potential conflict of interest.

## Publisher’s Note

All claims expressed in this article are solely those of the authors and do not necessarily represent those of their affiliated organizations, or those of the publisher, the editors and the reviewers. Any product that may be evaluated in this article, or claim that may be made by its manufacturer, is not guaranteed or endorsed by the publisher.
