# Corrigendum: Associations of Serum S100A12 With Severity and Prognosis in Patients With Community-Acquired Pneumonia: A Prospective Cohort Study

**DOI:** 10.3389/fimmu.2021.815903

**Published:** 2022-02-10

**Authors:** Xiao Jiang, Chun-Mei Huang, Chun-Mei Feng, Zheng Xu, Lin Fu, Xin-Ming Wang

**Affiliations:** ^1^ Department of Nephrology, First Affiliated Hospital of Anhui Medical University, Hefei, China; ^2^ Anhui Province Key Laboratory of Major Autoimmune Diseases, Anhui Institute of Innovative Drugs, School of Pharmacy, Anhui Medical University, Hefei, China; ^3^ Respiratory and Critical Care Medicine, Second Affiliated Hospital of Anhui Medical University, Hefei, China; ^4^ Department of Toxicology, Anhui Medical University, Hefei, China; ^5^ Department of Pharmacy, First Affiliated Hospital of Anhui Medical University, Hefei, China; ^6^ Third-Grade Pharmaceutical Chemistry Laboratory of State Administration of Traditional Chinese Medicine, Hefei, China

**Keywords:** S100A12, community-acquired pneumonia, CAP severity score, prognosis, biomarker

There is an error in the ethics name and number in the section of **Study Design and Subjects**. The correct ethics name and number is “Ethics Committee of Second Affiliated Hospital of Anhui Medical University (YX2021-085)”.

The authors apologize for this error and state that this does not change the scientific conclusions of the article in any way. The original article has been updated.

## Publisher’s Note

All claims expressed in this article are solely those of the authors and do not necessarily represent those of their affiliated organizations, or those of the publisher, the editors and the reviewers. Any product that may be evaluated in this article, or claim that may be made by its manufacturer, is not guaranteed or endorsed by the publisher.

