# Supplementary material for: Associations of Serum S100A12 With Severity and Prognosis in Patients With Community-Acquired Pneumonia: A Prospective Cohort Study
Source: Front Immunol. 2021 Oct 22;12:714026. doi: 10.3389/fimmu.2021.714026 (PMC8569229; doi:10.3389/fimmu.2021.714026)
Supplement: Supplementary file 1 [file Table_1.doc]

Supplemental Table 1. The predictive capacity of serum S100A12 and clinical characteristics for severity and death in CAP patients.

| Variables | Severity | | | | Death | | | |
| --- | --- | --- | --- | --- | --- | --- | --- | --- |
|  | AUC | Sensitivity | Specificity | *P* | AUC | Sensitivity | Specificity | *P* |
| S100A12 | 0.802 (0.704, 0.900) | 0.857 | 0.746 | ＜0.001 | 0.755 (0.606, 0.904) | 0.865 | 0.810 | 0.008 |
| CURB-65 | 0.884 (0.798, 0.969) | 0.643 | 0.986 | ＜0.001 | 0.768 (0.607, 0.929) | 0.727 | 0.885 | 0.006 |
| CRB-65 | 0.889 (0.804, 0.974) | 0.821 | 0.901 | ＜0.001 | 0.786 (0.630, 0.942) | 0.864 | 0.756 | 0.003 |
| PSI | 0.778 (0.666, 0.890) | 0.720 | 0.775 | ＜0.001 | 0.802 (0.644, 0.960) | 0.864 | 0.313 | 0.002 |
| CURXO | 0.795 (0.522, 0.954) | 0.643 | 0.704 | 0.020 | 0.717 (0.398, 0.835) | 0.901 | 0.788 | 0.227 |
| SMART-COP | 0.958 (0.912, 1.005) | 0.857 | 0.886 | ＜0.001 | 0.942 (0.886, 0.997) | 0.955 | 0.797 | ＜0.001 |
| APACHE Ⅱ | 0.795 (0.694, 0.895) | 0.607 | 0.859 | ＜0.001 | 0.760 (0.584, 0.935) | 0.591 | 0.963 | 0.007 |
| CRP | 0.595 (0.477, 0.7147) | 0.786 | 0.465 | 0.141 | 0.666 (0.507, 0.824) | 0.615 | 0.642 | 0.087 |
| IL-6 | 0.638 (0.511, 0.785) | 0.643 | 0.676 | 0.033 | 0.552 (0.315, 0.789) | 0.385 | 0.789 | 0.593 |
| TNF-α | 0.412 (0.270, 0.555) | 0.143 | 0.654 | 0.012 | 0.403 (0.130, 0.676) | 0.165 | 0.365 | 0.018 |
| MIP-2 | 0.602 (0.481, 0.728) | 0.964 | 0.239 | 0.007 | 0.549 (0.379, 0.720) | 0.231 | 0.412 | 0.009 |
| CURB-65+S100A12 | 0.916 (0.840, 0.992) | 0.821 | 0.910 | ＜0.001 | 0.794 (0.624, 0.964) | 0.905 | 0.872 | 0.002 |
| CRB-65+S100A12 | 0.932 (0.858, 1.006) | 0.924 | 0.862 | ＜0.001 | 0.810 (0.641, 0.979) | 0.905 | 0.820 | 0.001 |
| PSI+S100A12 | 0.846 (0.763, 0.930) | 0.758 | 0.892 | ＜0.001 | 0.851 (0.725, 0.976) | 0.905 | 0.673 | ＜0.001 |
| CURXO+S100A12 | 0.784 (0.689, 0.880) | 0.682 | 0.831 | ＜0.001 | 0.691 (0.485, 0.897) | 0.810 | 0.577 | 0.048 |
| SMART-COP+S100A12 | 0.959 (0.909, 1.009) | 0.894 | 0.982 | ＜0.001 | 0.943 (0.885, 1.000) | 0.809 | 0.769 | ＜0.001 |
| APACHE Ⅱ+S100A12 | 0.847 (0.758, 0.935) | 0.667 | 0.922 | ＜0.001 | 0.798 (0.622, 0.973) | 0.912 | 0.796 | 0.002 |
| CRP+S100A12 | 0.658 (0.535, 0.781) | 0.695 | 0.830 | 0.015 | 0.564 (0.371, 0.757) | 0.778 | 0.784 | 0.508 |
| IL-6+S100A12 | 0.813 (0.717, 0.909) | 0.687 | 0.735 | ＜0.001 | 0.763 (0.622, 0.904) | 0.687 | 0.821 | 0.007 |
| TNF-α+S100A12 | 0.588 (0.445, 0.730) | 0.583 | 0.916 | 0.176 | 0.597 (0.324, 0.870) | 0.833 | 0.801 | 0.318 |
| MIP-2+S100A12 | 0.792 (0.693, 0.891) | 0.727 | 0.741 | ＜0.001 | 0.743 (0.589, 0.897) | 0.664 | 0.794 | 0.012 |
